# Left-sided valvular heart disease and survival in out-of-hospital cardiac arrest: a nationwide registry-based study

**DOI:** 10.1038/s41598-023-39570-z

**Published:** 2023-08-04

**Authors:** Ellen Dejby, Deepak L. Bhatt, Kristofer Skoglund, Aidin Rawshani, Elmir Omerovic, Björn Redfors, Anna Myredal, Petur Petursson, Oskar Angerås, Arvid Gustafsson, Daniella Isaksén, Johan Herlitz, Araz Rawshani

**Affiliations:** 1 Department of Molecular and Clinical Medicine, University of Gothenburg, Institute of Medicine, Gothenburg, Sweden; 2 Mount Sinai Heart, Icahn School of Medicine at Mount Sinai, New York, NY USA; 3 Department of Cardiology, The Sahlgrenska University Hospital, Gothenburg, Sweden; 4 Clinical Trial Center, Cardiovascular Research Foundation, New York, NY USA; 5 Department of Cardiology, New York-Presbyterian Hospital/Columbia University Medical Center, New York, USA; 6 The Swedish Cardiopulmonary Resuscitation Registry, Centre of Registries, Västra Götaland, Gothenburg, Sweden

**Keywords:** Cardiology, Diseases, Health care

## Abstract

Survival in left-sided valvular heart disease (VHD; aortic stenosis [AS], aortic regurgitation [AR], mitral stenosis [MS], mitral regurgitation [MR]) in out-of-hospital cardiac arrest (OHCA) is unknown. We studied all cases of OHCA in the Swedish Registry for Cardiopulmonary Resuscitation. All degrees of VHD, diagnosed prior to OHCA, were included. Association between VHD and survival was studied using logistic regression, gradient boosting and Cox regression. We studied time to cardiac arrest, comorbidities, survival, and cerebral performance category (CPC) score. We included 55,615 patients; 1948 with AS (3,5%), 384 AR (0,7%), 17 MS (0,03%), and 704 with MR (1,3%). Patients with MS were not described due to low case number. Time from VHD diagnosis to cardiac arrest was 3.7 years in AS, 4.5 years in AR and 4.1 years in MR. ROSC occurred in 28% with AS, 33% with AR, 36% with MR and 35% without VHD. Survival at 30 days was 5.2%, 10.4%, 9.2%, 11.4% in AS, AR, MR and without VHD, respectively. There were no survivors in people with AS presenting with asystole or PEA. CPC scores did not differ in those with VHD compared with no VHD. Odds ratio (OR) for MR and AR showed no difference in survival, while AS displayed OR 0.58 (95% CI 0.46–0.72), vs no VHD. AS is associated with halved survival in OHCA, while AR and MR do not affect survival. Survivors with AS have neurological outcomes comparable to patients without VHD.

## Introduction

Valvular heart disease (VHD) is an increasingly common condition, with aortic stenosis (AS) being the most prevalent valvular lesion worldwide^1–3^. Left-sided valvular lesions are associated with high mortality and there are currently no effective medications that stall or reverse disease progression^4^. Risk factors for left-sided valvular lesions, aortic stenosis in particular, include age, high LDL cholesterol, high lipoprotein(a), obesity, hypertension, diabetes and renal dysfunction^5^. Individuals with left-sided valvular lesions typically exhibit significant comorbidities, particularly heart failure, coronary artery disease and atrial fibrillation^2^.

The 5-year mortality rate in AS is approximately 50%, making the condition as lethal as metastatic cancers^6^. Aortic regurgitation and mitral lesions are also associated with increased morbidity and mortality^7,8^. Left-sided valvular lesions, particularly aortic stenosis, are associated with increased risk of sudden cardiac arrest (SCA) and death (SCD). The annual incidence of SCD ranges from 0.3% to 3% in patients with aortic stenosis^9,10^, and one study showed that the cumulative incidence of SCD was 14% at 8 years^11^.

In the setting of SCA, all left-sided valvular lesions may render chest compressions ineffective due to the hemodynamic consequences of the stenosis or regurgitation (or both). Moreover, valvular lesions are typically accompanied by substantial comorbidities, and the presence of a left-sided valvular lesion may indicate that resuscitation will be futile. Indeed, a previous study of patients with in-hospital cardiac arrest (IHCA) showed that the probability of ROSC (return of spontaneous circulation) in patients with AS was 90% lower compared to patients without the condition^12^. Clinicians may be worried to initiate resuscitation in patients with left sided lesions given this pretext.

The extent to which left-sided valvular lesions affect survival in out-of-hospital cardiac arrest (OHCA) is unknown. We used the Swedish Registry for Cardiopulmonary Resuscitation (SRCR) to study the association between left-sided VHD and characteristics and survival in OHCA, with emphasis on aortic stenosis.

## Methods

### Study population

We used the SRCR to include all cases of OHCA during 2010 to 2020. The registry has been described previously^13^. The SRCR is a nationwide quality registry launched in 1990. All ambulance organizations in Sweden reported cases of OHCA to the registry during the study period. The registry employs Utstein style of reporting. We included all cases of OHCA where resuscitation was attempted, during the time period Jan 1st 2010 to Dec 31st 2020. The definition of OHCA in the registry is a cardiac arrest occurring outside of the hospital walls. Initial (presenting) rhythm is based on the first recorded electrocardiogram in the vast majority of cases, and interrogation of AED (automatic external defibrillators) reports in a minority if cases. Initial rhythm was categorized as ventricular fibrillation or pulseless ventricular tachycardia (VF/pVT), pulseless electrical activity (PEA) or asystole. Critical time intervals (delay from collapse to emergency call, no-flow time i.e. time from collapse to CPR, time to defibrillation, time to EMS dispatch and arrival) are reported.

### Data linkage

The Swedish Inpatient and Outpatient Registries are nationwide governmental databases that record all primary and secondary diagnoses recorded in inpatient and outpatient clinics throughout Sweden. The Inpatient Registry contains all inpatient records since year 1987 and has been validated^14^. The Outpatient Registry contains all outpatient clinic visits since 2002. The primary and up to 20 secondary diagnoses are available in each registry. Diagnoses are classified using the International Classification of Disease (ICD).

Medications were retrieved from the Swedish Prescribed Drug Registry, which includes all prescriptions filled since 2005. We retrieved prescriptions filled from 1st Jan 2008 according to Anatomical Therapeutic Chemical (ATC) classes.

The LISA (longitudinal integrated database for health insurance and labor market studies) database was used to obtain socioeconomic data, e.g. income, education, etc.

All these databases were merged with the SRCR using the unique 12-digit personal identification number assigned to all Swedish citizens.

### ICD-10 codes

Acquired aortic stenosis (AS) was defined as ICD-codes I350 or I352, aortic regurgitation (AR) as I351, mitral stenosis (MS) as I342, mitral regurgitation (MR) as I340 or I341. The first date for these diagnoses (in primary or secondary position) were retrieved from the Inpatient Registry. Patients who had been discharged with any of these diagnoses were classified as having the condition. Combined lesions were not assessed (grouping was based on the first valvular lesion recorded).

### Outcome measures

The primary outcome measure was survival at 30 days. The secondary outcome measure was neurological function measured using cerebral performance category (CPC) score. The CPC score was assessed at discharge and ranged from 1 to 5 (1, no sequelae; 2, mild sequelae; 3, severe sequelae; 4, vegetative state; 5, brain dead).

### Descriptive statistics

Patient characteristics are described using means, standard deviations, medians and interquartile ranges. No hypotheses tests were performed on baseline data since it includes the entire population with OHCA in Sweden during the time period. Coexisting conditions before and after diagnosis of each valvular condition was assessed for cardiovascular diagnoses (i.e. ICD chapters I00 to I99).

### Survival analysis

Time from diagnosis of VHD to cardiac arrest was evaluated using the Cox adjusted survival curves; adjustment was made for age and sex. Logistic regression was used to model 30-days survival as a binary outcome, and was adjusted for sex, age, location, no-flow time, and initial rhythm. Survival was assessed for each valvular lesion by using patients without VHD as the reference group. For each valvular lesion, we also calculated the relative variable importance^15^ of 450 candidate predictors. Variable importance is a measure of how important each predictor is, calculated using permutation accuracy importance. Hence, variable importance is measured as the difference in prediction accuracy before and after permuting the variable^16^. The purpose of this analysis was to evaluate whether determinants of survival differed across the groups.

All methods and procedures were executed in accordance with guidelines and regulations.

### Ethics approval

The study was approved by the Swedish Ethical Review Authority. All patients have provided their informed consent to participate in the registry.

## Results

We included 55,615 patients of whom 1948 had AS, 384 had AR, 17 had MS, and 704 had MR. Patients with MS will not be described henceforth due to the low number of cases.

### Characteristics prior to cardiac arrest

**Figure 1:**
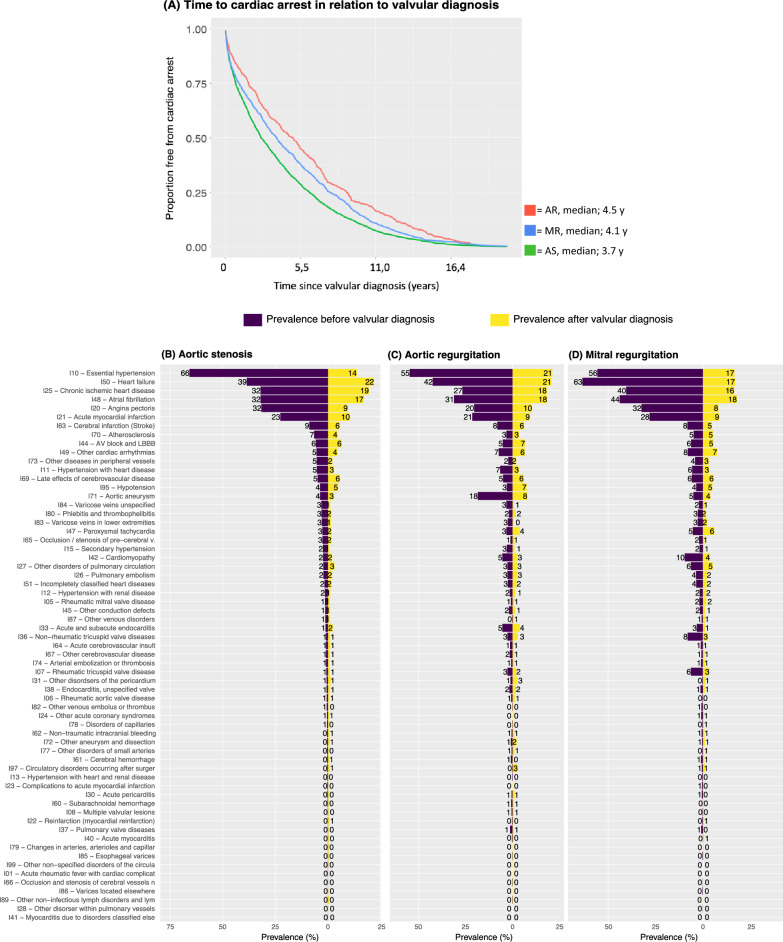
Cox adjusted survival curves from valvular diagnosis to cardiac arrest and coexisting and intervening conditions at valvular diagnosis. (**A**) Cox-adjusted survival curves for time from VHD to cardiac arrest. (**B**–**D**) Prior (i.e., to VHD diagnosis) and intervening (i.e., after VHD diagnosis) coexisting conditions. AS = aortic stenosis; AR = aortic regurgitation; MR = mitral regurgitation. Previous conditions is defined as comorbidities present prior to hospitalization for valvular disease. Intervening conditions refer to conditions developed after hospitalization for valvular disease, prior to cardiac arrest.

Figure 1A shows Cox adjusted survival curves (survival time depicts time from valvular diagnosis to cardiac arrest). The adjusted median time from valvular diagnosis to cardiac arrest was 3.7 years (1348.5 days) in AS, 4.5 years (1652.5 days) in AR and 4.1 years (1513 days) in MR. Figure 1B–D shows previous (i.e. prior to VHD diagnosis) and intervening (i.e. developed after VHD diagnosis, but before cardiac arrest) comorbidities. Hypertension was the most common condition prior to valvular diagnosis, being prevalent in 66% of patients who developed AS, 55% in AR and 56% in MR. Heart failure was more common prior to a diagnosis of MR (63%) and AR (42%) than AS (39%). Roughly one in five developed heart failure after being diagnosed with each respective valvular lesion. In all groups, the most common comorbidities developed after diagnosis of the valvular lesion were hypertension, heart failure, coronary artery disease and atrial fibrillation (Fig. 1B–D). Aortic aneurysm was roughly four times as common in patients with AR. Patients with MR had more atrial fibrillation and ischemic heart disease.

### Baseline characteristics at cardiac arrest

Roughly one third of all patients in all groups were female (Table 1). The mean age at cardiac arrest was 80.3 years for patients with AS, 73.6 years for patients with AR, 75.6 years for patients with MR and 68.3 years for patients without valvular disease. Cardiovascular etiologies were the underlying causes in 83.0% of patients with AS, 82.7% in AR, 84.1% in MR and 61.2% in those without valvular disease. With regards to clock time, cardiac arrest was most common between 7 am to 12 am in all groups, with roughly one in three cases occurring during that time (Table 1 and Supplementary Fig. 1). The vast majority of all cases occurred at home, with small differences across the groups.

**Table 1:** Baseline characteristics of patients with out of hospital cardiac arrest in relation to valvular heart disease.

	Aortic stenosis	Aortic regurgitation	Mitral stenosis	Mitral regurgitation	No valvular disease
n	1948	384	17	704	52,562
Female – n (%)	659 (33.9)	117 (30.5)	8 (47.1)	214 (30.4)	17,883 (34.1)
Age – mean (SD)	80.27 (10.19)	73.63 (13.73)	76.41 (10.98)	75.56 (12.29)	68.33 (18.07)
Cause of cardiac arrest – n (%)					
Cardiovascular disease	1440 (83.0)	278 (82.7)	10 (71.4)	528 (84.1)	28,560 (61.2)
Overdose / intoxication	2 (0.1)	3 (0.9)	0 (0.0)	2 (0.3)	1420 (3.0)
Trauma	15 (0.9)	2 (0.6)	0 (0.0)	8 (1.3)	1114 (2.4)
Pulmonary disease	80 (4.6)	5 (1.5)	1 (7.1)	22 (3.5)	2657 (5.7)
Suffocation	19 (1.1)	3 (0.9)	0 (0.0)	8 (1.3)	1256 (2.7)
Suicide	9 (0.5)	1 (0.3)	0 (0.0)	1 (0.2)	1110 (2.4)
Drowning	3 (0.2)	1 (0.3)	0 (0.0)	1 (0.2)	460 (1.0)
Sudden infant death syndrome	0 (0.0)	0 (0.0)	0 (0.0)	0 (0.0)	170 (0.4)
Other causes	167 (9.6)	43 (12.8)	3 (21.4)	58 (9.2)	9884 (21.2)
Clock time at cardiac arrest – n (%)					
0 to 6 am	291 (17.6)	54 (16.7)	2 (22.2)	90 (15.4)	7193 (16.4)
1 to 6 pm	446 (26.9)	91 (28.2)	0 (0.0)	181 (31.0)	13,297 (30.4)
7 to 11 pm	302 (18.2)	65 (20.1)	3 (33.3)	110 (18.8)	8367 (19.1)
7 to 12 am	617 (37.3)	113 (35.0)	4 (44.4)	203 (34.8)	14,872 (34.0)
Location of cardiac arrest – n (%)					
Home	1446 (74.5)	298 (78.0)	14 (82.4)	529 (75.7)	37,374 (71.4)
Public place	225 (11.6)	40 (10.5)	2 (11.8)	101 (14.4)	8621 (16.5)
Other places	270 (13.9)	44 (11.5)	1 (5.9)	69 (9.9)	6344 (12.1)
Critical time intervals, minutes – median (IQR)					
Time from arrest to CPR	3.00 [0.00, 10.00]	3.00 [0.00, 10.00]	4.00 [0.00, 14.00]	3.50 [0.00, 10.00]	3.00 [0.00, 10.00]
Time from arrest to EMS arrival	12.00 [8.00, 20.00]	12.00 [8.00, 20.00]	18.50 [13.00, 23.50]	12.00 [8.00, 19.00]	13.00 [8.00, 20.00]
Time from arrest to defibrillation	17.00 [10.00, 26.00]	14.00 [9.00, 24.00]	19.50 [16.50, 23.25]	15.50 [10.00, 23.00]	15.00 [8.00, 24.00]
Time from arrest to ROSC	15.00 [8.00, 21.00]	13.00 [8.50, 20.00]	23.00 [19.75, 34.25]	15.00 [10.00, 23.00]	15.00 [9.00, 23.00]
Witnessed cardiac arrest – n (%)	1348 (70.9)	243 (65.0)	9 (56.2)	433 (63.6)	32,890 (64.5)
Bystander treatment – n (%)	957 (50.9)	194 (53.2)	8 (50.0)	344 (51.0)	27,988 (55.3)
Bystander defibrillation – n (%)	17 (32.7)	6 (60.0)	0 (NaN)	5 (26.3)	624 (36.2)
Initial rhythm – n (%)					
VF/pVT	365 (21.2)	92 (26.7)	5 (33.3)	199 (31.8)	10,713 (23.1)
PEA	428 (24.8)	75 (21.8)	1 (6.7)	114 (18.2)	7803 (16.8)
Asystole	932 (54.0)	177 (51.5)	9 (60.0)	313 (50.0)	27,817 (60.0)
Consciousness on EMS arrival – n (%)	230 (12.1)	48 (12.7)	0 (0.0)	65 (9.5)	5385 (10.5)
Pulse on EMS arrival – n (%)	280 (15.1)	69 (18.9)	0 (0.0)	75 (11.3)	6960 (14.0)
Mechanical compressions – n (%)	797 (42.5)	166 (45.0)	7 (41.2)	284 (42.1)	20,232 (40.2)
Intubation – n (%)	505 (26.4)	100 (26.8)	3 (17.6)	183 (26.6)	14,646 (28.4)
Defibrillation, any – n (%)	665 (35.2)	140 (38.0)	6 (35.3)	306 (45.0)	16,695 (33.1)
Number of defibrillations – mean (SD)	3.33 (2.90)	4.01 (3.91)	4.60 (2.88)	4.53 (3.77)	3.46 (3.15)
Adrenaline given – n (%)	1558 (80.9)	307 (81.2)	12 (75.0)	554 (79.4)	40,859 (78.7)
Amiodarone given – n (%)	236 (12.4)	59 (15.8)	3 (18.8)	141 (20.4)	5926 (11.6)
Coronary angiography performed – n (%)	6 (27.3)	5 (55.6)	0 (NaN)	4 (40.0)	279 (37.4)
PCI performed – n (%)	35 (14.8)	15 (19.5)	0 (0.0)	17 (12.5)	3218 (30.0)
Coexisting conditions – n (%)					
Hypertension	1549 (79.5)	284 (74.0)	13 (76.5)	482 (68.5)	22,493 (42.8)
Heart failure	1173 (60.2)	234 (60.9)	10 (58.8)	565 (80.3)	10,687 (20.3)
Ischemic heart disease	991 (50.9)	161 (41.9)	4 (23.5)	401 (57.0)	9948 (18.9)
Atrial fibrillation	955 (49.0)	187 (48.7)	14 (82.4)	448 (63.6)	9759 (18.6)
Diabetes	680 (34.9)	66 (17.2)	6 (35.3)	183 (26.0)	9674 (18.4)
Arthrosis	489 (25.1)	87 (22.7)	4 (23.5)	133 (18.9)	7997 (15.2)
Dyslipidemia	698 (35.8)	87 (22.7)	7 (41.2)	203 (28.8)	7704 (14.7)
Stable or unstable angina pectoris	798 (41.0)	104 (27.1)	4 (23.5)	275 (39.1)	7311 (13.9)
Colorectal cancer	380 (19.5)	75 (19.5)	2 (11.8)	131 (18.6)	7353 (14.0)
Alcohol, overuse	182 (9.3)	58 (15.1)	1 (5.9)	82 (11.6)	7616 (14.5)
Acute myocardial infarction	612 (31.4)	104 (27.1)	3 (17.6)	239 (33.9)	6579 (12.5)
Medications – n (%)					
Anticoagulants, antiplatelet agents (B01)	1288 (66.1)	254 (66.1)	13 (76.5)	439 (62.4)	18,132 (34.5)
Beta-blockers (C07)	1143 (58.7)	239 (62.2)	8 (47.1)	429 (60.9)	16,821 (32.0)
ACEi/ARB (C09)	898 (46.1)	207 (53.9)	6 (35.3)	414 (58.8)	16,674 (31.7)
Diuretics (C03)	1092 (56.1)	175 (45.6)	7 (41.2)	425 (60.4)	13,321 (25.3)
Lipidlowering drugs (C10)	789 (40.5)	128 (33.3)	7 (41.2)	271 (38.5)	12,026 (22.9)
Calcium channel blockers (C08)	411 (21.1)	70 (18.2)	5 (29.4)	89 (12.6)	8263 (15.7)
Antidiabetic drugs (A10)	448 (23.0)	39 (10.2)	4 (23.5)	112 (15.9)	7697 (14.6)
Antibiotics	373 (19.1)	83 (21.6)	4 (23.5)	122 (17.3)	7148 (13.6)
Cardiac drugs in C01	532 (27.3)	74 (19.3)	4 (23.5)	202 (28.7)	5804 (11.0)

VF/pVT = ventricular fibrillation/pulseless ventricular tachycardia; ACEi = angiotensin converting enzyme inhibitor; ARB = angiotensin receptor blocker; PCI = percutaneous coronary intervention; CPR = cardiopulmonary resuscitation; EMS = emergency medical service (ambulance); am = ante meridiem; pm = post meridiem; ROSC = return of spontaneous circulation.

Median time from cardiac arrest to start of CPR was 3 min for AS, 3 min for AR, 3.5 min for MR and 3 min for those without valvular disease. Time to first defibrillation was 17 min for AS, 14 min for AR, 15.5 min for MR and 15 min for those without VHD. Time to EMS arrival was 12 min for AS, AR, MR and 13 min for those without VHD. Time to ROSC was 15, 13, 15, 15 min for AS, AR, MR, and those without VHD, respectively.

The initial rhythm was VF/pVT in 21.2% in AS, 26.7% in AR, 31.8% in MR, and 23.1% in patients without VHD. With regards to comorbidities (at the time of cardiac arrest) in patients with AS, 79.5% had hypertension, 60.2% had heart failure, 50.9% had chronic coronary artery disease, 49.0% had atrial fibrillation, 34.9% had diabetes, 35.8% had dyslipidemia.

Table 2 presents crude survival parameters. Return of spontaneous circulation (ROSC) at hospital arrival was observed in 34.6% of patients with AS, 41.7% in AR, 44.9% in MR and 45.1% in patients without VHD. Roughly 9% of patients with AS, AR and MR were conscious at hospital arrival, compared with 11% in patients without valvular disease (missing rate was high, as presented in Table 2). ROSC at any time occurred in 27.5% with AS, 33.3% with AR, 36.4% with MR and 34.7% in those without VHD. Survival at 30 days was 5.2% in AS, 10.4% in AR, 9.2% in MR and 11.4% in those without VHD. One-year survival was 3.9%, 8.1%, 8.1% and 10.2% in AS, AR, MR and no VHD, respectively.

**Table 2:** Outcomes in patients with out of hospital cardiac arrest in relation to valvular heart disease.

	Aortic stenosis	Aortic regurgitation	Mitral stenosis	Mitral regurgitation	No valvular disease
ROSC at hospital arrival (missingness: ~ 45%)	347 (34.6)	95 (41.7)	2 (33.3)	182 (44.9)	13,564 (45.1)
ROSC at any time (missingness ~ 5%)	513 (27.5)	124 (33.3)	3 (23.1)	242 (36.4)	17,352 (34.7)
Conscious at hospital arrival (missingness ~ 50%)	89 (9.0)	20 (9.0)	1 (16.7)	36 (9.1)	3272 (11.1)
Alive at 30 days (missingness ~ 0%)	101 (5.2)	40 (10.4)	0 (0.0)	65 (9.2)	5985 (11.4)
Alive at 365 days (missingness ~ 0%)	75 (3.9)	31 (8.1)	0 (0.0)	57 (8.1)	5382 (10.2)
Alive until end of follow-up (missingness ~ 0%)	49 (2.5)	26 (6.8)	0 (0.0)	39 (5.5)	4323 (8.2)

ROSC = return of spontaneous circulation.

### Rates of ROSC, hospitalization and 30 days survival in relation to no-flow time

**Figure 2:**
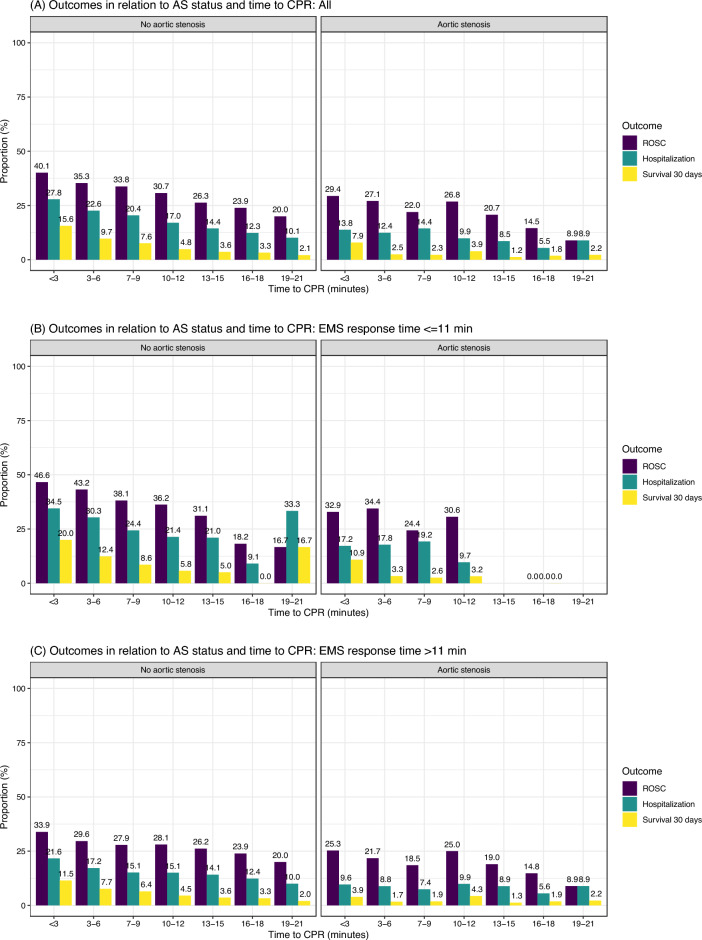
Rates of ROSC, hospitalization and 30 days survival in relation to aortic stenosis status. Crude rates of ROSC, hospitalization and 30-days survival in patients with AS and those with no VHD.

Figure 2 shows rates of ROSC, hospitalization and survival at 30 days in relation to no-flow time. Rates of ROSC, hospitalization and 30-days survival were clearly lower in people with AS compared to those without AS. ROSC rates declined from 40.1% when CPR was started in < 3 min to 20.0% when CPR was started in19-20 min in people without AS. Corresponding figures for those with AS were 29.4% and 8.9%, respectively. Survival rates for people with AS were very low throughout. For the majority (56%) of patients with AS, the delay to CPR was ≥ 3 min; survival was 1.2% to 3.9% in these patients. In patients where delay to EMS arrival was more than 11 min, survival was 11.5% in those without AS and 3.9% in those with AS when CPR was started in < 3 min. Supplementary Fig. 2 shows ROSC, hospitalization and survival rates in people with AS in relation to initial rhythm. As evident, there were virtually no survivors in people with AS presenting with asystole or PEA when no-flow time was longer than 10 min. Supplementary Figures 3 through 6 shows corresponding figures for AR and MR.

### Short- and long-term survival after cardiac arrest

**Figure 3:**
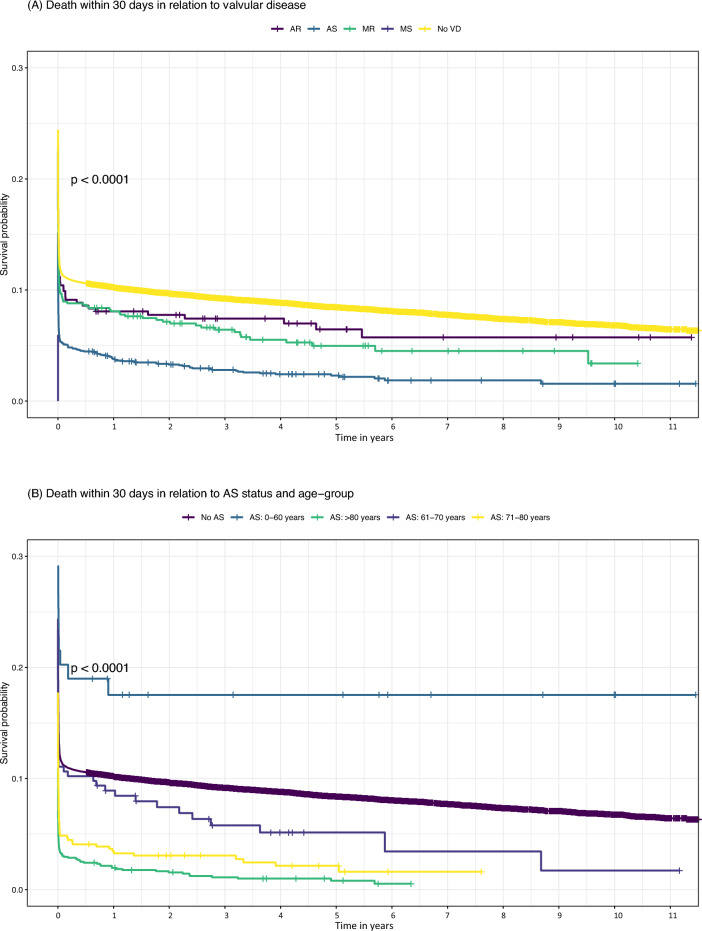
Kaplan Meier curves for survival from cardiac arrest. Kaplan–Meier curves describing short- and long-term survival stratified by (**a**) VHD status and (**b**) age-group in patients with AS. Note that y-axes are truncated.

Short- and long-term survival was highest for patients without VHD (Fig. 3A). Survival dropped to around 10% for AR and MR within the first few days, but thereafter survival tended to be higher in those with AR. All 17 patients with MS died on the first day. Patients with AS had the poorest survival among the other left-sided valvular lesions. Figure 3B shows Kaplan–Meier curves for patients with AS in relation to age.

### Neurological outcomes (cerebral performance)

**Figure 4:**
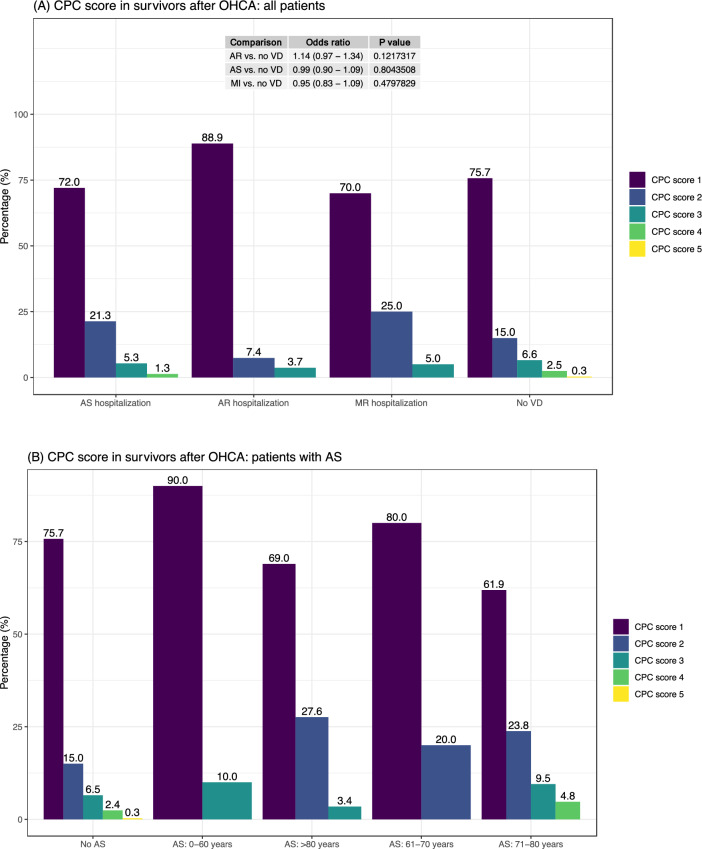
Cerebral performance category score. Distribution of CPC score in relation to (A) VHD status and (B) age-group in patients with AS. Odds ratios are adjusted for age and sex.

Figure 4A–B shows CPC scores among survivors. CPC score 1 (no neurological sequalae) was observed in 72.0% of patients with AS, 88.9% of patients with AR, 70.0% of patients with MR and 75.7% of patients with no valvular disease. Odds ratios, adjusted for age and sex, for surviving with CPC score 1, did not differ in AS, AR nor MR (all compared with no VHD). In patients of age 61–70 years with AS, no survivor had CPC score worse than 2.

### Adjusted probability of survival

**Figure 5:**
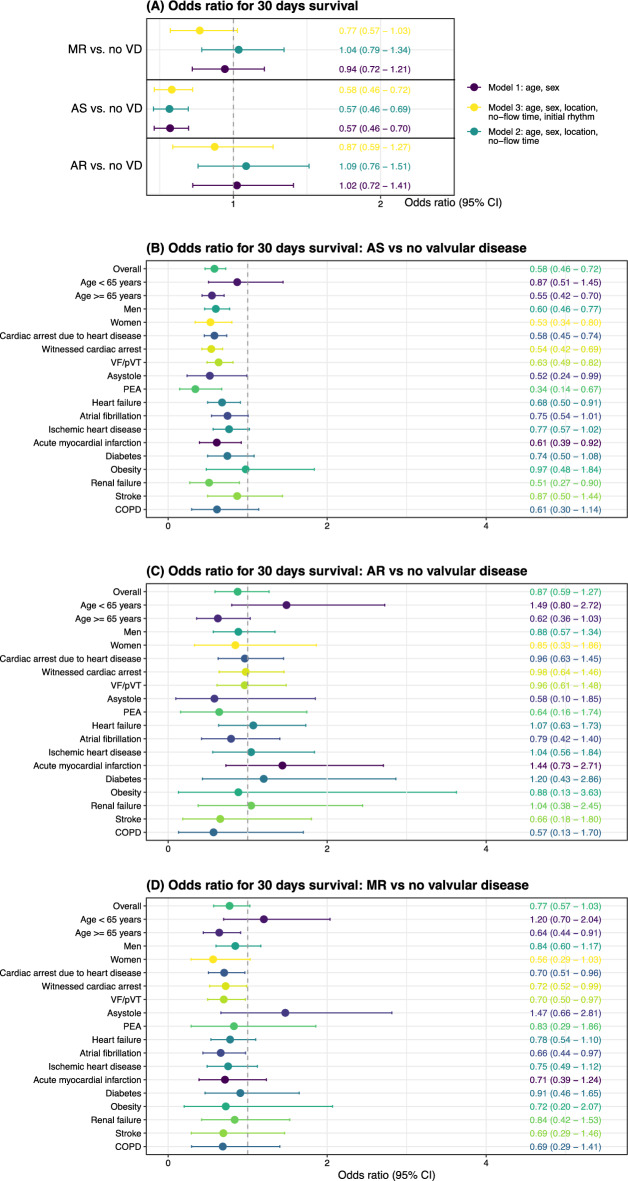
**A–D** Adjusted probability of survival overall and in subgroups. AS = Aortic stenosis. AR = Aortic regurgitation. MR = Mitral regurgitation. Logistic regression for 30-days survival in the (**A**) overall cohort and subgroups (**B**–**D**). Models in (**B**–**D**) were adjusted for age, sex, initial rhythm, location, and no-flow time.

Figure 5A–D shows probability of survival overall in each valvular group and in 18 subgroups respectively (Fig. 5B–D).

Overall odds ratios (Fig. 5A) were sequentially adjusted. As compared with no VHD, those with MR and AR showed no statistically significant difference in survival, while patients with AS displayed odds ratios of 0.57 in model 1 (95% CI 0.46–0.70), 0.57 in model 2 (95% CI 0.46–0.69) and 0.58 in model 3 (95% CI 0.46–0.72).

The association between AS (Fig. 5B) and 30-days survival was not significantly modified by age, sex, witnessed status, or coexisting conditions. However, the lowest odds ratio (judging the point estimates) of survival was noted in the subgroup of patients presenting with PEA, who displayed an odds ratio of 0.34 (95% CI 0.14–0.67).

With regards to AR, no subgroup displayed any significant difference in 30 days survival, although confidence intervals were wide (Fig. 5C).

For MR (Fig. 5D), the majority of all point estimates were below 1.0, suggesting a lower probability of survival. While in the overall analysis, there was no difference in survival between MR and no VHD, we note that for patients aged ≥ 65 years, those with cardiac etiology, VF/pVT, and patients with atrial fibrillation, survival was worse (~ 30% lower probability) in patients with MR, compared to no VHD.

### Relative importance of predictors of survival

There were no significant differences with regards to determinants of survival in AS, AR and MR (Supplementary Fig. 7).

## Discussion

At the heart of resuscitation lies the aim to maximize flow through the left ventricular outflow tract, in order to generate critical cerebral and myocardial perfusion pressure until circulation can be restored. Left-sided valvular lesions, AS in particular, presumably reduces the efficiency of chest compressions. AS is the most common valvular lesion in the Western world and no previous study has elucidated the association between AS and survival in OHCA. We present the largest study to date on AS, AR, MS and MR in OHCA, including a total of 3053 patients with left-sided valvular lesions, and 52,562 patients without VHD.

The main finding of our study is that patients with AS had 42% lower probability of survival, compared to those without VHD, and patients with AS presenting with PEA had 66% lower probability of survival, compared to patients without valvular lesions. However, patients with MR and AR showed no difference in survival compared to patients without lesions. Yet, neurological outcome among survivors with AS did not differ compared to other patients. Our data suggests that since patients with AS who survive OHCA have neurological function on a par with those without VHD, the former group should not be excluded from resuscitation attempts. We also demonstrate that the time from diagnosis of the valvular lesion to cardiac arrest is considerably shorter in AS (3.7 years) than in AR and MR (4.5 years and 4.1 years, respectively), which suggests that AS is more prone to cardiac arrest, although this conclusion should preferably be derived from analyses including patients who do not develop cardiac arrest.

A previous study investigated 51 patients with AS who developed an in-hospital cardiac arrest^12^. It was reported that patients with aortic stenosis had 90% lower probability of sustained ROSC, 86% lower probability of survival to discharge and 84% lower probability of survival with CPC score 1 or 2. We did not observe such a dismal prognosis in a sample with 1948 cases of AS who experienced an OHCA. Thus, while we report a lower survival in patients with AS, the survival in our cohort is considerably better as compared with the study on IHCA. Furthermore, that study also reported a graded association between degree of aortic stenosis and survival, which provide some evidence to our hypothesis.

It is also important to note that MR and AR were fairly prevalent but had no impact on survival. It is difficult to explain these findings since we expected valvular insufficiency to also result in diminished cerebral and myocardial perfusion, ultimately leading to worse outcomes. Thus, while MR and AR have a negative hemodynamic effect in subjects whose stroke volumes depend on ventricular contractions, it can be speculated that the driving forces of circulation during resuscitation may be less sensitive to valvular insufficiency, although this is highly speculative. Furthermore, it is evident that MS is very rare in this setting, with only 17 cases being recorded over a period of 10 years of nationwide cardiac arrest monitoring.

We also note that patients with AS may be even more time sensitive than patients without AS (Fig. 2). As time to CPR increases, survival in patients without AS decreases gradually from 15.6% (at < 3 min delay to CPR) to 2.1% (at 19–21 min delay to CPR). For patients with AS on the other hand, survival was at 2.5% already at 3–6 min delay to CPR. However, we observed unexpected findings with regards to crude survival in relation to time to CPR and EMS response times, with survival being higher when CPR started late and the EMS arrived later. We believe this is due to selection bias, with EMS personnel selecting to continue resuscitation attempts in cases with conditions perceived as favorable upon their arrival at the scene (e.g. when observing high quality bystander CPR). Overall survival was, however, lower with increasing EMS response time.

This study clearly demonstrates that patients with AS who present with PEA or asystole have virtually no chance of survival (Supplementary Fig. 2), regardless of no-flow time. This could serve as a valuable clinical predictor when managing patients with OHCA and a history of hospitalizing for aortic stenosis; those presenting with PEA or asystole will most likely die.

The fact that patients with AS have lower survival but exhibit no difference in neurological function among survivors suggests that those with AS and very poor prognosis die, leaving the stronger patients to contribute to CPC scores. This was evident from crude survival rates which was 5.2% in AS and 11.4% in patients without valvular lesions.

We also examined whether there was any subgroup, among 18 selected subgroups, for whom the association between AS and survival differed, as compared with the overall estimate. The only clear difference was the one noted for patients with PEA, who displayed very poor survival. We did not note any significant variations for AR, nor for MR.

Neither did we find any difference with regards to relative importance of a very large number of predictors (n = 460) of survival. This is clinically important as it suggests that the same management and focus can be applied to all patient groups studied here, without affecting probability of survival. Initial rhythm, age, early circulatory signs and critical time intervals are of pivotal importance, dwarfing the effect of all comorbidities, management, medications used, socioeconomic status, etc.

### Strength and limitations

This is the largest study to date examining the impact of left-sided valvular lesions on survival in OHCA. With regards to level of ascertainment, the Inpatient Registry is complete and records every single case discharged with any of the ICD-10 codes used in the current study. However, using ICD-10 codes, we are unable to grade the severity of the valvular lesion (which would require echocardiographic parameters). This is a limitation of the current study. Therefore, the coefficients obtained represent the average population effect (i.e. the overall average association between the lesion and the outcome). It is likely that those with severe lesions (particularly severe AS and MS) drive the estimates. Of note, approximately 60% of the patients with AS had heart failure, which supports that these were not just patients with mild AS noted incidentally on an echocardiogram. Moreover, the same limitation applies to the routinely used definition of heart failure, namely used ICD-9 and ICD-10 codes for heart failure hospitalization, which does not take severity or systolic or diastolic function into account^14,17–19^. Also, we did not have data on surgical or transcatheter aortic valve replacement, which are interventions that are likely to improve survival in patients with AS. Finally, it is important to note that misclassification may occur in both groups. Cases classified as non-AS cases may actually have undiagnosed AS. Such cases are more likely to represent less severe lesions that cause fewer symptoms. However, this study demonstrates the association of known lesions at the timing of cardiac arrest.

## Conclusions

We show that AS is the left-sided lesion most rapidly resulting in cardiac arrest, and survival in those with aortic stenosis is roughly halved, albeit neurological outcomes among survivors with AS is comparable to those without the lesion. Resuscitation should not be withheld patients with aortic stenosis, although those presenting with PEA or asystole have a very dismal prognosis. Aortic regurgitation and mitral regurgitation seem not to have any material effect on survival in OHCA and should therefore not affect decisions to withhold or initiate resuscitation attempts.

### Supplementary Information

Supplementary Information.

## Data Availability

The data that support the findings of this study are available from SRCR, The Swedish Inpatient and Outpatient Registry, The Swedish Prescribed Drug Registry, and The LISA database. Restrictions apply to the availability of these data, which were used under license for the current study, and so are not publicly available due to ethical reasons.
