# Supplementary material for: Left-sided valvular heart disease and survival in out-of-hospital cardiac arrest: a nationwide registry-based study
Source: Sci Rep. 2023 Aug 4;13:12662. doi: 10.1038/s41598-023-39570-z (PMC10403503; doi:10.1038/s41598-023-39570-z)

Supplementary Figure 1. Circadian variation in time of cardiac arrest in relation to valvular lesion

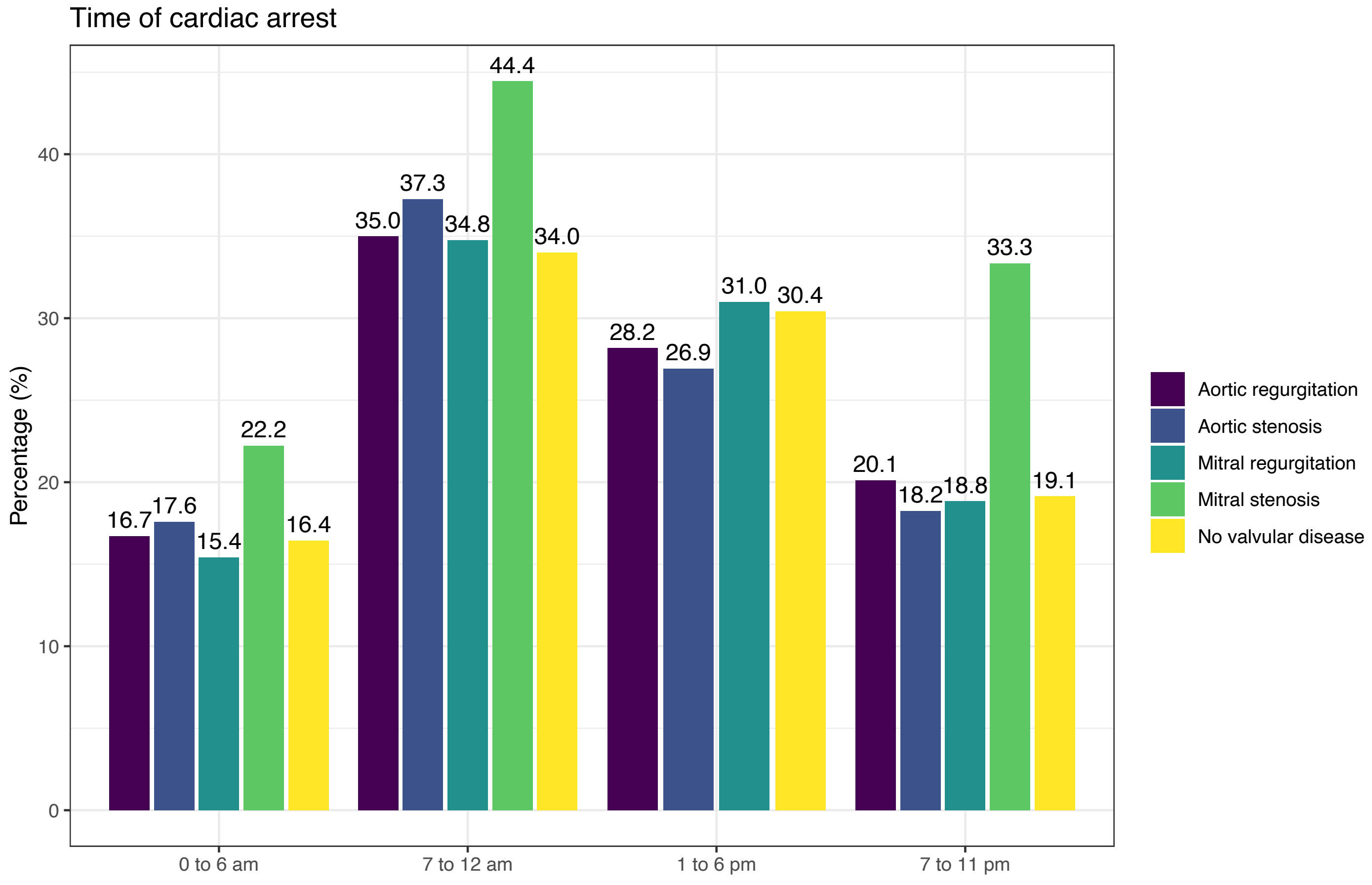

Supplementary Figure 2

(A) Outcomes in relation to inital rhythm (only patients with AS) and time to CPR: All

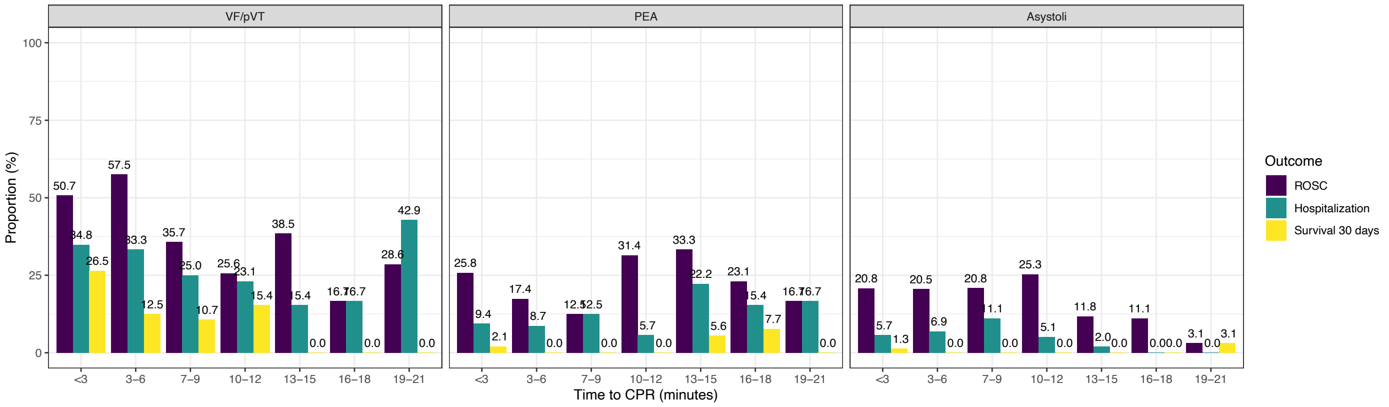

Supplementary Figure 3

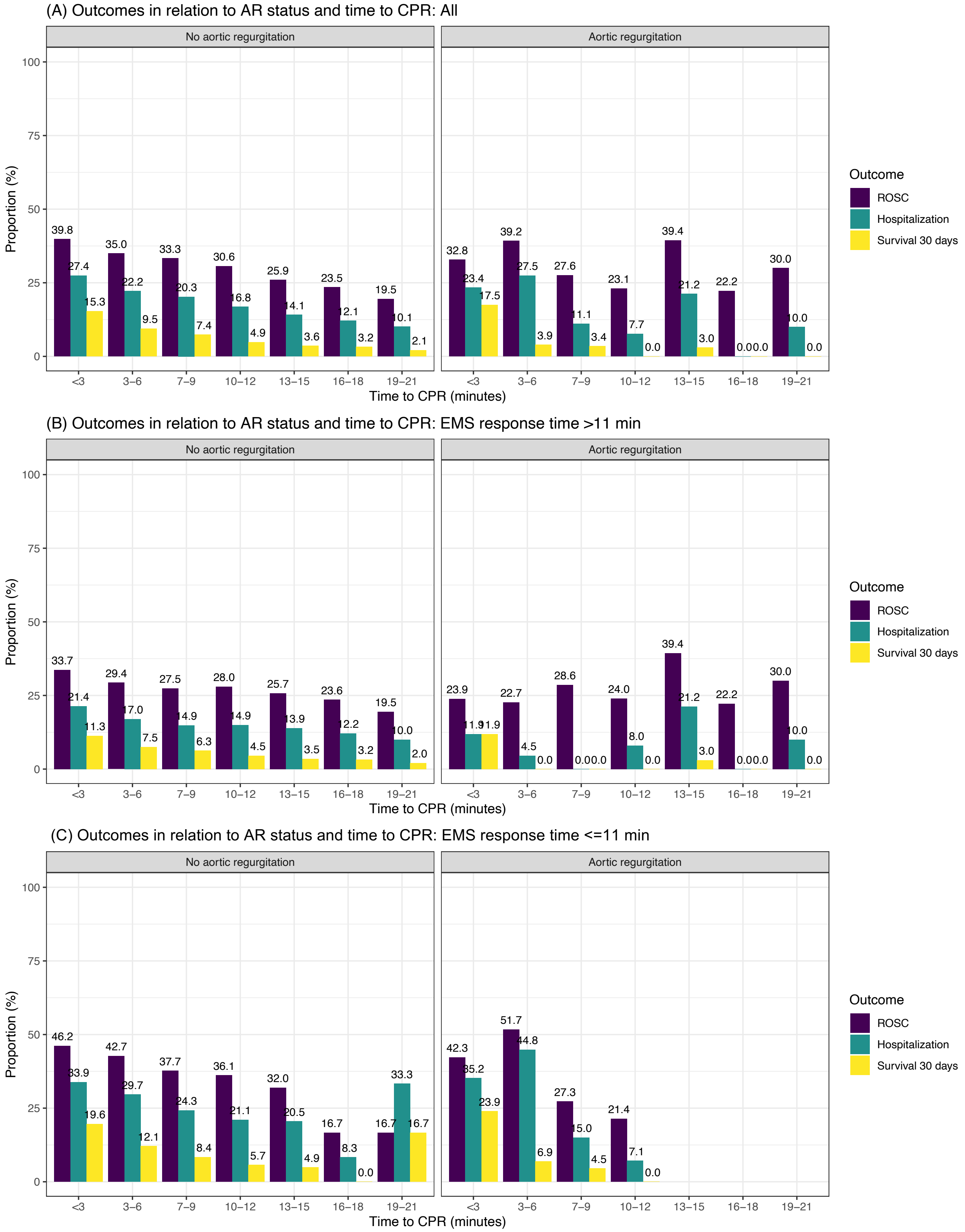

Supplementary Figure 4

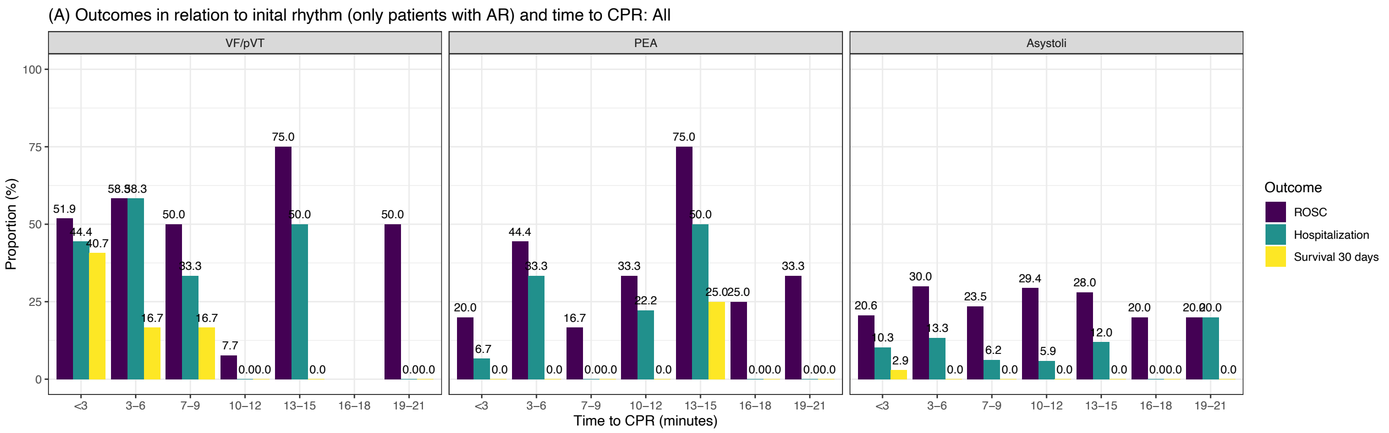

Supplementary Figure 5

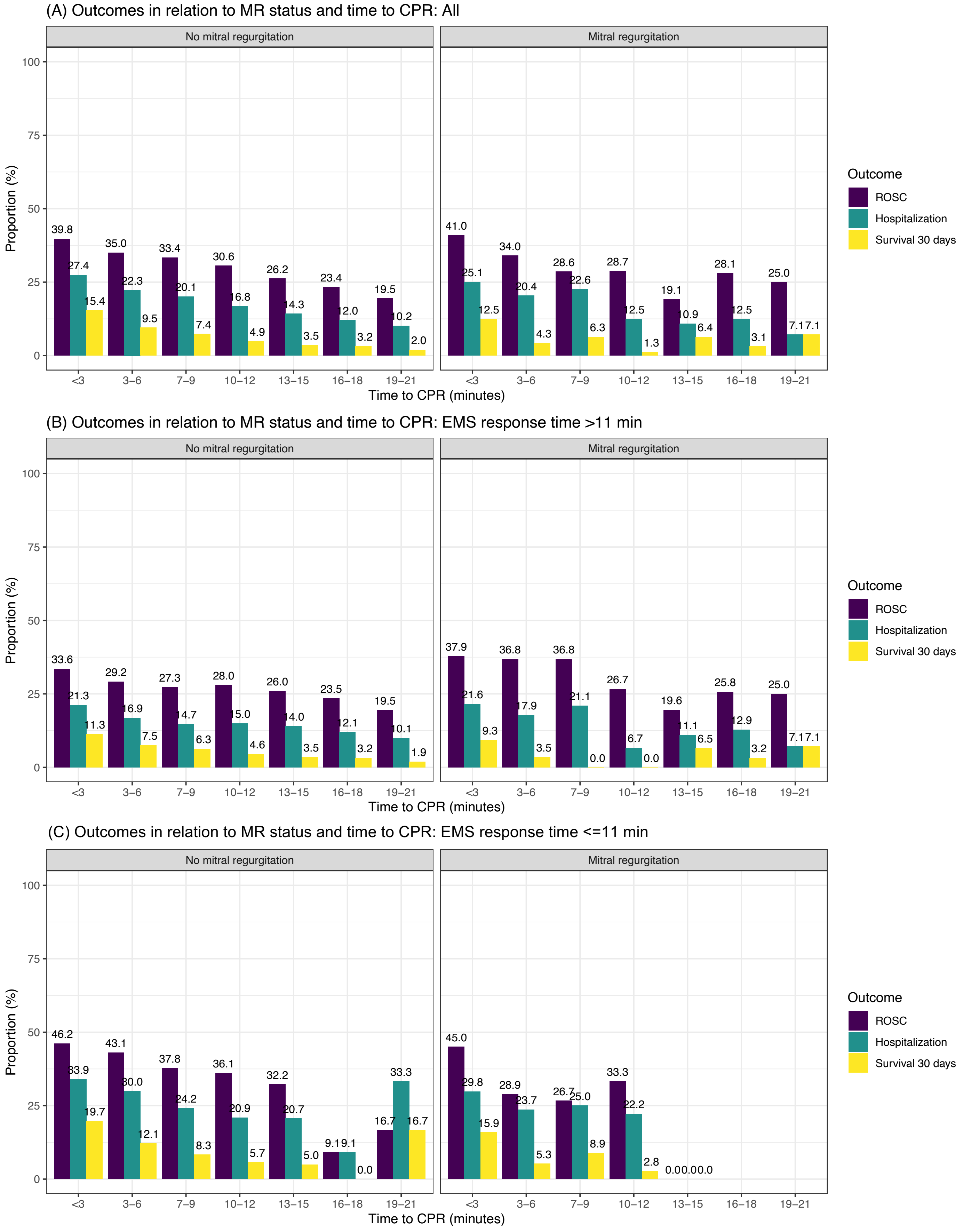

Supplementary Figure 6

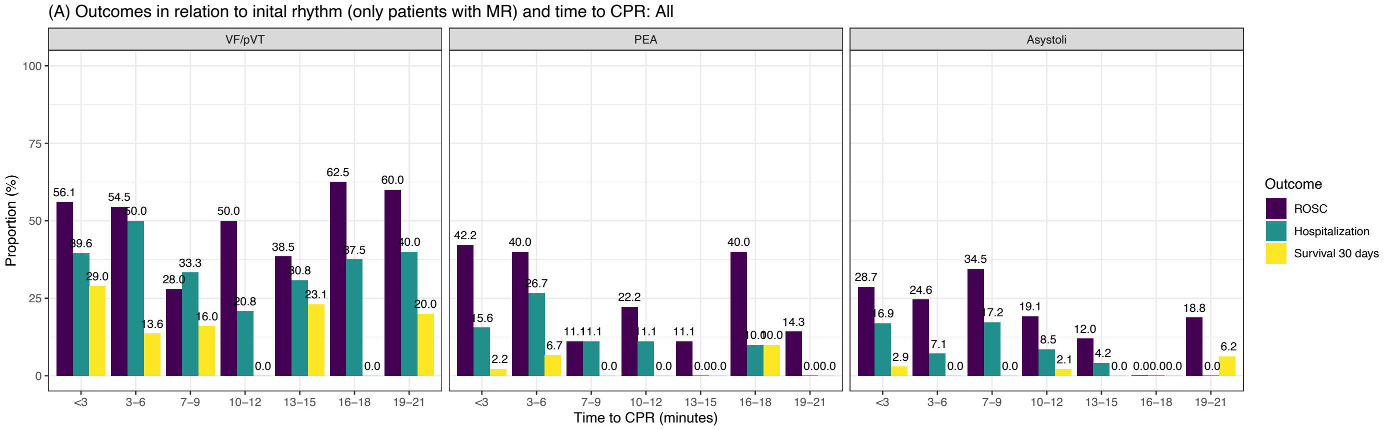

Supplementary Figure 7. Relative importance of predictors of survival

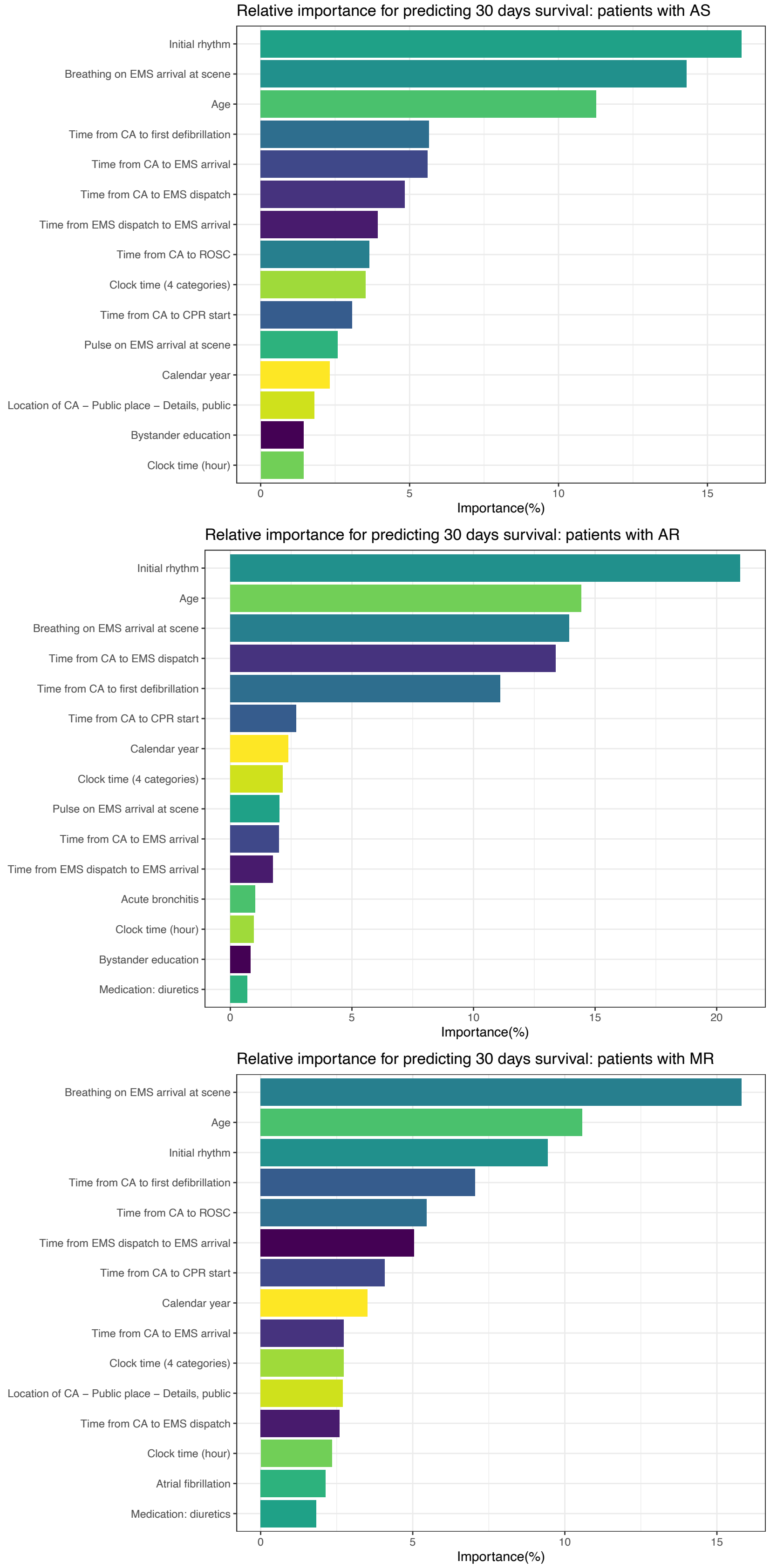

Supplement: Supplementary file 1 — Supplementary Information. [file 41598_2023_39570_MOESM1_ESM.pdf]
